# An Examination of COVID-19-Related Stressors among Parents

**DOI:** 10.3390/ejihpe11030061

**Published:** 2021-08-06

**Authors:** Sarah Alonzi, Jae eun Park, Angélica Pagán, Courtney Saulsman, Madison W. Silverstein

**Affiliations:** 1 Department of Psychology, Tulane University, New Orleans, LA 70118, USA; jpark14@tulane.edu; 2 Department of Psychology, Loyola University, New Orleans, LA 70118, USA; ampagan@my.loyno.edu (A.P.); cbsaulsm@my.loyno.edu (C.S.); mwsilver@loyno.edu (M.W.S.)

**Keywords:** COVID-19, stress, family, gender, health status, parenting

## Abstract

The circumstances of the COVID-19 pandemic have taken a psychological toll on parents. Thus, understanding the impact of these contextual stressors on parents is important to help inform the development of family-based health promotion interventions. The present study examined parents’ perception of various sources of stress resulting from the COVID-19 pandemic. Participants (N = 294) completed an open-ended question about their primary source of stress during the pandemic, which we coded into one or more of the following categories: family, work, health, and finance. We used chi-square tests to determine whether gender, marital status, financial strain, and education level were significantly related to each of the four primary sources of stress. We found that female, married, and financially strained participants were more likely to report family-related stressors. Further, we found that participants who expressed concern over health-related stressors were more likely to have pre-existing health conditions. Finally, we found that single participants were more likely to express concerns over financial stressors. Our findings shed light on parental concerns following the pandemic and inform new research directions, clinical approaches, and policy issues at the individual, community, and societal levels.

## 1. An Examination of COVID-19-Related Stressors among Parents and Recommendations to Mitigate Concerns at the Individual, Community, and Policy Levels

The turbulent effects of the coronavirus (SARS-CoV-2) has caused sudden and profound levels of psychosocial stress for families and children across the world [1]. In late 2019, the World Health Organization (WHO) declared the pandemic a global public health emergency [2], and as of January 2021, more than 100 million people worldwide had been infected with COVID-19 (Centers for Disease Control and Prevention [3]. The rapid rate of contagion and patterns of transmission have threatened individuals’ perceptions of control and safety, increasing levels of stress in their everyday lives. As the number of COVID-19 cases surged, protective policy measures such as government-mandated lockdowns and social distancing guidelines were enforced to mitigate the spread of the virus and reduce the burden on the health system [4]. 

Although these measures helped reduce the spread of disease, they have also resulted in abrupt closures of businesses, schools, and workplaces, affecting millions of lives at the individual, occupational, and the societal level. Unfortunately, these measures disrupted families’ financial and psychological stability and exacerbated economic and social disparities [5]. In particular, the stress exerted by the pandemic has had substantial effects on parental—and subsequently children’s—psychological well-being. Even before the pandemic, parenting daily hassles [6,7] were the norm for most families. These stressors may involve children misbehaving, or the abundance of labor-intensive tasks associated with parents’ caregiving routine [8,9]. In either case, these hassles are common in many households and often shared across multiple contexts (e.g., socioeconomic status, cultural backgrounds), thus the typical daily hassle of parenting may be of little significance in and of itself [7].

During the pandemic however, the impact of such relatively minor events may accumulate to a greater source of stress for both the children and the parents. Previous research has shown that chronic experience of these events may influence the quality of parenting, the parent–child relationship, and even child functioning [10,11]. Weeks after the World Health Organization declared COVID-19 to be a pandemic, 1.38 billion children worldwide were out of school or childcare [12], and uncertainty surrounding in-person schooling continues today [13]. Due to closures of schools and childcare centers [14], working parents are dealing with the increased demands of homeschooling with little to no preparation, and simultaneously meeting the expectations of their jobs as they attempt to work remotely. Online learning has also been a source of concern for parents, with parental depression and stress being negatively associated with their perceived preparation for assisting with at-home education needs for their children [15]. 

Further, closures of community facilities (e.g., playgrounds [12]) has increased the challenge of keeping children occupied and safe at home [16]. These additional responsibilities result in increased burnout, stress, and exhaustion [17,18], particularly for women (citation withheld for blind review), making it even more difficult for parents to effectively handle these challenges. Additionally, the modification in structure and the reduced opportunities for families to enjoy social and physical activities has potential long-term developmental consequences for children [19], and such disruption in daily routines can be particularly detrimental for young and school-aged children with limited coping strategies [9].

Early adolescence, though less studied, is another key period that may present a myriad of challenges for parents during the pandemic. Previous studies have found an increase in mental illnesses such as anxiety, depression, and traumatic symptoms [15] as well as an increase in adolescents with eating disorders resulting in hospitalization [20]. Further, studies reported greater conflict and reduced warmth and relationship quality among parents and adolescents during COVID-19 [21,22]. In addition to the unprecedented disruptions and restrictive measures caused by the pandemic, this may also be attributed to the increase in proximity with parents [23,24], which in turn may result in an increase in the frequency or intensity of cycles of discord in the home [25]. The challenges generated by COVID-19 are exacerbated for parents with teenage children, since adolescence is a particularly vulnerable period in life with a myriad of developmental needs [26]. 

There are also contexts outside of the regular family system that may affect parents’ perceived stress levels, most notably the parents’ workplace. Previous studies suggest that greater work-related stress and financial issues are associated with reduced sensitivity, responsiveness and patience towards family members [27,28]. Further, Wadsworth and Compas [29] found that high levels of economic strain are associated with increases in marital conflict. Indeed, the pandemic has had profound effects on the financial and economic well-being of families. Since April 2020, more than 30 million people have been furloughed [30]. The resultant strain on financial circumstances has had devastating impact on families as they experienced difficulties sustaining basic needs, such as paying rent and securing food and reliable childcare [31,32]; Past research has shown that socioeconomic disadvantages, such as unemployment and financial stress, are associated with poor mental health outcomes including symptoms of depression, anxiety, and psychosomatic symptoms, and low self-esteem [33]. Further, financial strain has been shown to have an adverse influence on parenting practice and children’s well-being, increasing their risk for developing problem behaviors [34,35]). 

Ongoing concerns regarding one’s own or one’s family members’ safety have also been frequently cited as a contextual stressor [36,37,38]). In a study by Dyregrov et al. [39], a large proportion of individuals expressed serious concern about the safety of their family members. These concerns may increase the psychological stress in parents, and an inability to cope effectively with these concerns may result in severe psychological distress, particularly for parents with pre-existing physical and mental health difficulties (citation withheld for blind review). 

In his seminal paper, Belsky [40] asserted that such contextual sources of stress play a prominent role in determining the influence of parenting on family functioning. Thus, these findings suggest the need to examine multiple contextual processes that affect parents’ perceptions of stress and its subsequent impact on the family system as a whole. An abundance of studies has examined the impact of stressful circumstances such as financial difficulties (e.g., Waylen et al., 2010 [41]) or interparental conflict (e.g., Gong et al., 2016 [42]) on parenting in isolation. However, during the COVID-19 pandemic, these stressors are concurrent and sustained, making it a unique, multi-dimensional stressor. Therefore, the objective of the present study was to identify the primary COVID-19-related stressors among parents. Based on previous research, we hypothesized that stressors related to childcare, relationships, physical health, mental health, work, and finances would emerge. 

We also aimed to examine how these stressors varied by demographic characteristics (i.e., gender, marital status, financial strain, health status, and education level) to identify parents at the highest risk for difficulties. Hypotheses were largely exploratory. However, we hypothesized that mothers would be more concerned about childcare, single parents and individuals with financial strain would be more concerned about finances, and individuals with pre-existing health conditions would be more concerned about physical health. Finally, we aimed to provide tailored suggestions at the individual, community, and policy levels for reducing family stress and increasing resources for families to promote resilience among children during the pandemic, as well as to inform future research and practice.

## 2. Method

### 2.1. Participants and Procedure

Participants were identified from a larger study of attitudes and behaviors about the COVID-19 pandemic (N = 1572). Participants were recruited using snowball sampling via advertisements on various social media platforms such as Facebook, Reddit, and Twitter. After consent was obtained, the participants completed surveys via Qualtrics online survey platform. Qualtrics is a useful tool for internet research studies because the embedded Javascript can be programmed to prevent missing data [43]. Data collection occurred from April 2020 to mid-May 2020. All procedures were approved by the university institutional review board. To ensure data quality, responses were evaluated through a Qualtrics embedded data feature. This allowed us to exclude repetitive responses (n = 68) and remove participants with questionable answers in terms of validity to the open-ended questions (i.e., such as malicious or nonsensical responses; n = 12) from the larger sample.

Participants were included in the present study if they reported having at least one child under the age of 18 years old. Based on these criteria, 294 participants, who ranged in age from 20 to 67 years old (M = 38.25, SD = 7.01) were included in this study. 

### 2.2. Measures

Participants’ stressors during the pandemic were reported using the Stress Mindset Measure-Specific (SMM-S); [44]. Participants responded to the open-ended SMM-S question asking, “What is the primary source of stress in your life right now?”.

### 2.3. Demographics

Information about participants’ age, race, ethnicity, marital status, health status, financial status, education level, and number and ages of children was collected using an investigator-developed demographics questionnaire. 

### 2.4. Data Analytic Plan

Upon careful review of each response to the open-ended question on the SMM-S, the senior author identified four broad categories that encompassed the primary sources of stress in participants’ lives during the pandemic. Subsequently, two independent raters (the first and second authors) carefully reviewed and coded each response into one or more of the four categories. After evaluating the interrater reliability between the independent raters, any disagreements were resolved by discussion between the two raters. Participants were prompted to answer the survey item in the provided textbox before continuing with the survey and, thus, no data were missing. 

Analyses were conducted using SPSS (Version 27). Inter-rater reliability for the four categories was assessed using Cohen’s Kappa. We used chi-square tests with an alpha-level of 0.05 to determine whether gender, marital status, health status, financial strain, and education level were significantly related to each of the four categories of primary source of stress. 

## 3. Results

### 3.1. Participant Characteristics 

The vast majority of participants (83%) were from the United States. Participants outside of the United States were from Canada (9%), Australia (2%), the United Kingdom (5%), and elsewhere (1%). The majority were White (86%), female (56%), married (80%), possessed a bachelor’s degree or higher (65%), reported no high-risk physical health conditions (58%), and were not financially strained (70%). Most participants had one (46%) or two (40%) children under the age of 18 years old. The average age of children under 18 living in each household was 7.33 (SD = 4.83). 

### 3.2. Perceived Stressors 

Four categories of stressors emerged: children and family (44%), work (29%), health (18%), and financial (17%). The two raters demonstrated strong inter-rater reliability as measured by Cohen’s Kappa in classifying participant responses into the established categories: children and family (κ = 0.88, *p* < 0.001), work (κ = 0.75, *p* < 0.001), health (κ = 0.74, *p* < 0.001), and financial (κ = 0.77, *p* < 0.001). 

### 3.3. Family-Related Stressors 

Of each of the categories, children and family was mentioned by the most participants. This category included any reference to difficulty facilitating distance learning (e.g., “trying to keep the kids on a schedule and interested in learning.”), entertaining children (e.g., “My 3-year-old being so sedentary and the fact that we are probably destroying her life by living like this.”), childcare inaccessibility (e.g., “the lack of knowing if there will be childcare for my school-aged child. Many of the programs have been shut down or have no solid plans yet.”), or alterations to day-to-day household duties and family life (e.g., “Trying to do everything all the time all at home all together. Parenting, being a spouse, working, general chores, and tasks and doing none of it well.”). Participants who expressed concern over children and family were more likely to be women, χ2 (1, N = 294) = 5.45, *p* = 0.020), married, χ2 (1, N = 294) = 7.95, *p* = 0.005), and financially strained, χ2 (1, N = 294) = 6.301, *p* = 0.012). Notably, there were no significant differences in frequency of concerns about children and family between those who were and were not college educated, χ2 (1, N = 294) = 0.713, *p* = 0.400, or between individuals with and without pre-existing health conditions, χ2 (1, N = 294) = 2.63, *p* = 0.104. 

### 3.4. Work-Related Stressors 

The second most commonly expressed concern was regarding work, which included any mention of job insecurity (e.g., “not knowing if my job will come back in June.”), work-life balance or burnout (e.g., “work-life balance while working at home with two kids.”), and worker’s rights violations (e.g., “spouse’s job violating his employment contract. A need to move for a new job.”). 

Participants who expressed concern over work-related stressors were more likely to not have pre-existing health conditions, χ2 (1, N = 294) = 4.31, *p* = 0.040, and to have at least a bachelor’s degree, χ2 (1, N = 294) = 4.03, *p* = 0.045. There were no significant differences in frequency of concern for work-related stressors by marital status, χ2 (1, N = 294) = 0.230, *p* = 0.630, gender, χ2 (1, N = 294) = 0.622, *p* = 0.430, or financial strain, χ2 (1, N = 294) = 1.21, *p* = 0.271.

### 3.5. Health-Related Stressors 

The next commonly reported stressor was health related. This category included testing inaccessibility (e.g., “feeling sick and not able to get tested.”), health endangerment due to negligent response to the pandemic (e.g., “the thought of my son’s daycare reopening anytime soon while cases are not declining,” and “how many people will die that didn’t need to because we opened too early?”), mortality concerns (e.g., “not wanting to die.”), concern over routine appointments in healthcare settings (e.g., “worrying about ongoing healthcare if hospitals become overwhelmed. I don’t want to attend doctor’s offices right now.”), and concerns for high-risk family and friends (e.g., “worrying about my parents and grandmother… everyone’s health if they were to get it.”). 

Participants who expressed concern over health-related stressors were more likely to have pre-existing health conditions, χ2 (1, N = 294) = 5.15, *p* = 0.023. There were no significant differences in frequency of health-related concerns by gender, χ2 (1, N = 294) = 0.044, *p* = 0.834, marital status, χ2 (1, N = 294) = 1.531, *p* = 0.216, education level, χ2 (1, N = 294) = 3.440, *p* = 0.064, or financial strain, χ2 (1, N = 294) = 2.380, *p* = 0.123. 

### 3.6. Financial Stressors 

The least frequently recounted concern was financial, with responses including disparaging attitudes towards capitalism (e.g., “leaving my home to participate in the asinine rat-race of late-stage Capitalism.”), lack of government stimulus (e.g., “no money. No government support.”), and unplanned or unfavorable financial decisions (e.g., “I’m not behind on my credit card payments, but I’m just paying the minimum, which accrues interest…”). 

Participants who expressed concerns over financial-related stressors were more likely to be single, χ2 (1, N = 294) = 5.35, *p* = 0.021, and experience financial strain, χ2 (1, N = 294) = 25.61, *p* < 0.001. There were no significant differences in frequency of financial concerns by gender, χ2 (1, N = 294) = 0.188, *p* = 0.665, education level, χ2 (1, N = 294) = 0.839, *p* = 0.360, or presence or absence of health conditions, χ2 (1, N = 294) = 3.12, *p* = 0.077.

## 4. Discussion

This present investigation demonstrated that common stressors for parents of at least one child under the age of 18 can be categorized into four broad groups of concerns surrounding family, work, health, and finances. These findings are consistent with other qualitative research, which identified prevalent response domains of COVID-19-related stressors, including financial difficulties, physical health, and children’s wellbeing and academics [1]. We found that individual-level differences in gender, marital status, education level, presence or absence of physical health conditions, and financial strain were associated with differences in many of these four broad concerns. Specifically, family and children-related concerns were most common within women, married, and non-financially strained individuals. As hypothesized, we found that women more commonly reported concerns over children and family-related stressors than men. Moreover, work concerns were most common among those with pre-existing health conditions and a college education; health concerns were most common among those with pre-existing conditions; and financial concerns were most common among financially strained individuals and single parents. These findings illuminate distinct patterns in concerns during the pandemic and have the potential to inform efforts to ease the burden of the pandemic for families through swift action and intervention at the individual, community, and policy levels. 

Although stress has considerably increased for parents of children under 18, generally [45], these concerns were more common among women, married individuals, and those without financial strain. Women might be more likely to express concern over children and families because women are generally more involved in childcare and household duties [46,47]. Our finding that married participants were more concerned about family-related stressors is unexpected, given that single-parent households likely face more challenges without the ability to divide increased responsibilities with a spouse [15]. However, married participants, who are likely spending a significantly increased amount of time with their spouses, might face role confusion over online schooling and other household tasks [47], leading to increased conflict and concern for children. Lastly, those without financial strain were more likely concerned about family stressors, likely due to financial strain often being the dominant concern for low SES families [48]. 

As family and children-related stressors are common, multifaceted solutions, both targeted toward mental health and policy-level pandemic management are necessary to ameliorate these concerns within families, and particularly mothers. At the individual-level, we recommend increasing access to teletherapy for individual parents or spousal partners who are struggling with shifted household roles and facilitating distanced learning, as teletherapy outcomes have been shown to be similar in efficacy to in-person outcomes [49]. Additionally, developing coping strategies appropriate for heightened-stress, such as stress reappraisal [50] and mindfulness based stress reduction, and utilization of mobile phone mindfulness applications might reduce parents’ distress during the pandemic. Further, there is support for the efficacy of family-based interventions via telehealth to support parenting behaviors and caregiver well-being [51]. However, it should also be noted that technology-assisted interventions are less effective in socially disadvantaged populations due to limited access to technology, so it would be essential to ensure that preexisting inequities in access to care are not exacerbated [52]. 

At the community level, As uncertainty regarding the modality of children’s school was frequently mentioned, transparency within schools detailing education modality plans and contingencies are necessary [53]. At the policy level, the facilitation of a safe return to schools has the potential to significantly reduce stress for parents. However, this cannot occur without governmental action providing the necessary resources to allow students and teachers to utilize protective equipment, facilitate distanced recreational activities, and mandated vaccination [3]. Of course, throughout the pandemic, there has been rampant anti-vaccine sentiment and vaccines are not available and approved for pediatric use in many countries. Further, there are disparities in access to vaccines internationally, which adds complexity to vaccinating children. Acknowledging these factors, we suggest that parents of school-aged children are provided with informative and accurate vaccine guidance from schools and pediatricians, which are tailored to the unique vaccine circumstances in their countries. In addition, as the majority of caregiving duties often fall on women and might seriously detract from non-caregiving jobs, unemployment benefits could be expanded to cover those leaving their primary employment to provide childcare amid the pandemic [54]. However, it is worth noting that such policies might further remove women from the workforce, which could be detrimental given that the majority of those who became unemployed during the pandemic were women [55,56]. Thus, balancing policies focusing on supporting child-rearing and workforce reentry is necessary to ensure that women are completely supported. 

Concerns over work-related stressors particularly affected those without pre-existing health conditions and those with a bachelor’s degree or higher. Those with no health comorbidities might be asked to return to work earlier which can be more stressful, whereas those with health conditions might be able to provide documentation to delay the return to in-person work, alleviating stress. To support individuals with and without health comorbidities, wherever possible, employers should attempt to provide employees with the option to work from home or in-person, depending on preference and local disease transmission at the time. Those without bachelor’s degrees might be more likely to be essential workers, meaning that individuals with bachelor’s degrees could more frequently report stress due to the transition to remote work. To support those transitioning to work at home, employers can promote adaptive mindsets towards remote work (e.g., the idea that remote work is a skill that can be developed), as fixed mindsets (e.g., the belief that an individual is immutably not suited for remote work), which are associated with negative affect and perceived reduction in productivity in individuals transitioning to remote work [57]. For those continuing to work in-person, union requests for government-subsidized personal protective equipment, particularly for those in high public contact industries (e.g., healthcare, retail, food service), might attenuate stress [58].

Health-related concerns were unsurprisingly more prevalent among parents with pre-existing health conditions; however, action to combat medical aversion, psychologically informed public health initiatives, and governmental action are required. Allaying fears of contracting COVID-19 in medical facilities among the general population is imperative, as such fears cooccurring with widely publicized health system overburden can lead to a vast array of public concerns likely to outlast the immediate effects of the pandemic. Such concerns include women close to giving birth skipping antenatal appointments [59], a general decline in cancer screening [60] and vaccination [61], and reproductive health services inaccessibility [62]. To address these fears and prevent the potentially deleterious effects of avoiding essential health services and appointments, hospitals and other medical facilities can initiate campaigns within their communities to emphasize safety protocols and implement stringent masking and distancing requirements to protect patients [63]. Patients can be sent appointment reminders more frequently, along with memorable details about the increased safety and low risk of disease transmission at medical facilities. In order to provide this quality of care and safety, particularly over a prolonged period of time, governmental action is required, and future aid should be allocated to hospitals based on COVID-19- burden as opposed to hospitals’ 2019 fee-for-service revenues, which was how the Coronavirus Aid, Relief, and Economic Security (CARES) Act allocated support to hospitals [64]. Finally, to mitigate health disparities among low SES families, which could be exacerbated by inability to utilize telehealth services, electronic devices must be allocated to patients who lack financial resources [65]. 

Financial stressors were most prevalent among unmarried parents and, by a very large effect, those who reported financial strain. Our null findings for gender differences are consistent with Barzilay et al. [66] who found that men and women were comparably worried about the financial impact of the virus. The finding that unmarried parents more frequently cited financial concerns as a perceived stressor is consistent with the notion that financial strain is among the foremost contributors to stress in single parents [67]. Notably, a qualitative analysis published prior the COVID-19 pandemic advised behavioral health professionals working with single parents to be knowledgeable about single parents’ unique responsibilities within the household and actively assist in the identification and acquisition of resources and sources of support [68]. Our results indicate that this recommendation is even more important during the COVID-19 pandemic. 

Although financial concerns might be a common fear throughout the pandemic, those who faced financial distress prior to the pandemic are likely to face much greater consequences [69]. Among the prominent reasons for financial strain during the pandemic is inadequate governmental relief incentives [69]. Government relief in the form of direct aid, particularly in the form of monthly stimulus payments to citizens, is much needed to ease the burden on families during the pandemic, especially those with a single parent or pre-existing strain [70]. As unemployment in the United States has been met with inadequate governmental support [71], expanding unemployment eligibility and allocation might further alleviate stress. 

The present study possesses several noteworthy strengths and limitations. A major strength was the use of an open-ended question, which allowed participants to freely express stressors allowing for diverse answers. In accordance with these stressors, we offer tangible solutions at the individual, community, and policy levels to support families, broadly, as well as targeted solutions for subgroups that might be the most vulnerable to a particular stressor. Despite these strengths, these findings are limited by the predominantly white, non-Hispanic sample, which is not representative of the global population. To address this limitation, future studies should attempt to recruit a representative participant sample, which might offer greater insight into demographic determinants of financial stressors [72]. We acknowledge that these policy recommendations are most applicable to North America and Western countries, which reflects the geographic composition of our sample. Thus, these suggestions should be interpreted with the understanding that geographic areas are affected differently by the pandemic and there is no universal policy suggestion that can assist parents globally, without tailoring based on disease transmission and public health measures, mental health resources, and school factors. Additionally, the sample was relatively homogeneous in terms of marital status and education level, with the majority of participants possessing a bachelor’s degree. Moreover, we did not collect data on participants’ gender and sexual identity. As LGBTQIA+ parents might face pandemic stressors’ unique to their gender or sexual identity, future research should query participants on these topics and recruit a diverse sample to allow for subgroup analyses to examine the role of gender and sexual identity in pandemic stressors. Future research would also benefit from including those with more representative educational attainment. Finally, we assert that this was an exploratory analysis of cross-sectional data. Thus, these results should be interpreted with caution, as it is not possible to assume causality and factors beyond the original scope of this assessment, such as mental health status or gender and sexuality, might confound these associations. Despite the limitations, these findings outline tangible suggestions to mitigate the adverse effects of the pandemic on the well-being of parents, which can be modified depending on geographic and political factors and researched further in more representative samples internationally. 

## 5. Conclusions

As scientists, we are uniquely positioned to investigate the best ways to build resiliency in families amidst major systemic changes that can undermine family functioning. The results of this study indicate that parents with at least one child under the age of 18 faced four broad concerns surrounding children and family, work, health, and finances. Gender, marital status, education level, physical health conditions, and financial strain were associated with differences in many of these four broad concerns. At the individual level, recommendations include teletherapy, stress reappraisal, mindfulness application utilization, and considering remote work to be a skill to be developed. At the community level, we encourage local hospital initiatives to focus on safety features to allay safety concerns that prevent hospital visitation. At the policy level, we encourage interdisciplinary collaboration within hospitals to facilitate resource accessibility, governmental economic stimulus, return-to-work flexibility for those with pre-existing health conditions, and unambiguous guidelines for public schools to attenuate parent confusion over schooling. These findings can illuminate those in the most need of intervention and promote the utilization of necessary resources to assist families.

## Data Availability

Research data are not shared.
